# Hip Hemiarthroplasty in Patients with Multiple Myeloma: A Retrospective Case Series and Review of the Literature

**DOI:** 10.1055/s-0044-1792099

**Published:** 2025-01-16

**Authors:** Ahmed Nageeb Mahmoud, Alejandro Ordas-Bayon, Catherine Mary Doyle, Maria F. Echeverry-Martinez, Daniel S. Horwitz

**Affiliations:** 1 Departamento de Cirurgia Ortopédica, Geisinger Medical Center, Danville, PA, Estados Unidos; 2 Departamento de Cirurgia Ortopédica, Ain Shams University, Cairo, Egito; 3 Departamento de Cirurgia Ortopédica, Hospital Universitario Ramon y Cajal, Madri, Espanha

**Keywords:** femoral neck, hemiarthroplasty, hip fractures, multiple myeloma

## Abstract

**Objectives** Femoral neck fractures in multiple myeloma patients are usually managed with hemiarthroplasty or total hip arthroplasty, depending on the presence of acetabular infiltration. Due to the paucity of dedicated studies, the aim of the present study is to review the clinical outcomes of hip hemiarthroplasty in patients with multiple myeloma and to review the literature regarding the outcomes and survival in these patients' subset.

**Methods** There were 15 patients (16 cases), with a mean age of 71.7 years, who had myeloma and received hip hemiarthroplasty for displaced femoral neck fractures had their radiographs and clinical data assessed for this study. From those, 13 cases received bipolar and 3 unipolar hemiarthroplasty.

**Results** After a mean follow-up of 18.2 months since the time of surgery, 3 cases suffered periprosthetic fractures (18.75%), 4 cases (25%) had heterotopic ossification, and 1 case (6.25%) had acetabular erosion. The 1- and 5-year patient mortality rates for the study cohort were 53.3 and 73.3%, respectively.

**Conclusion** Hip hemiarthroplasty remains a viable treatment option in myeloma patients; however, the potentially high morbidity and mortality in these patients should be sensibly understood before the surgery to achieve satisfactory expectations.

## Introduction

Plasma cell dyscrasias represent a wide range of conditions, including monoclonal gammopathy of undetermined significance (MGUS; Kyle disease), solitary plasmacytoma of bone, and multiple myeloma. 1 Multiple myeloma is the most common primary bone malignancy and typically presents with fatigue, musculoskeletal pain, pathologic fractures, or recurrent infection. 1 2 3 The pelvis and proximal femur are common sites of osteolytic infiltrations caused by solitary or multiple myeloma lesions. These lesions usually present with pain and impending or pathological fractures. 4 5 Surgical management of femoral neck fractures in myeloma patients includes either hemiarthroplasty or total hip arthroplasty, depending mainly on the presence of acetabular involvement. 3 4 6

The outcomes of prosthetic replacement in metastatic bone disease have been well studied in previous literature. However, no study has specifically evaluated the outcomes of hemiarthroplasty in myeloma fracture cases. The available studies have either reported collectively on the results of two or more of the treatment options, being open reduction and internal fixation (ORIF), hemiarthroplasty (HA) and total hip arthroplasty (THA) in myeloma, 4 6 or reported on a cohort that included myeloma and other non-myeloma causes of bone infiltration. 7 8 9 10 11 12 13 14 15 The aim of the present study is to report our outcomes in a series of hemiarthroplasty cases in patients with myeloma performed at a level-1 trauma center in an effort to add to the existing scarce evidence. Our main study question was whether myeloma patients would have high incidence of complications, particularly fractures and acetabular wear after HA. We also aim to provide a literature review that includes all reported hemiarthroplasty case series in myeloma patients that have been reported in the literature.

## Materials and Methods

After Institutional Review Board approval, a retrospective study was performed to evaluate all hemiarthroplasty patients, using the Current Procedural Terminology (CPT - American Medical Association, Chicago, IL, USA) codes. Between January 1988 and June 2023, a total of 2,488 HA cases were identified. All the cases were reviewed against our inclusion and exclusion criteria to extract the cases and data relevant for this study. The data collected included patients' demographics, clinical information, and radiographic evaluation. Detailed information about the follow-up and postoperative clinical course were collected for all cases.

### Inclusion Criteria

1 - Patients who underwent HA for a hip fracture, either primarily after trauma or for management of femoral neck fracture nonunion.
2 - Patients who had a diagnosis of multiple myeloma or plasmacytoma of bone.

### Exclusion Criteria

1 - Patients without clinical follow-up notes after HA surgery.

## Results

A total of 18 cases (17 patients) of plasma cell dyscrasias that underwent HA for femoral neck fractures have been retrieved from our database. Two patients have been excluded due to having non-progressive MGUS, leaving 16 cases (15 patients) for inclusion in this study.

Of these patients, 9 were female and 6 were male, with a mean age of 71.7 years (range: 39.2–89.6) at the time of primary HA surgery. One female had bilateral femoral neck fractures, which occurred 2 months apart and received bilateral cemented unipolar HA. Fourteen patients (15 cases) had multiple myeloma, and 1 patient had a solitary bone plasmacytoma at the time of fracture that progressed to multiple myeloma later. Apart from plasma cell dyscrasias, 10 patients (11 cases) had additional significant, frequently combined medical comorbidities as shown in Table 1 . The mean body mass index (BMI) for 12 patients was 32.9 (range: 22–52.2), while in 3 other patients, data regarding BMI was not available in their records.

**Table 1:** Medical comorbidities in the study patients

Chronic kidney disease	4 (1 ESRD)
Diabetes mellitus	2
Heart failure	2
Severe or morbid obesity (BMI ≥ 35)	3
Hypothyroidism	1
Parkinson disease	1

Abbreviations: BMI, body mass index; ESRD, end-stage renal disease.

All of the cases had been diagnosed with femoral neck fractures secondary to low-energy trauma (fall while walking). Nine patients had been diagnosed with multiple myeloma prior to presentation with femoral neck fractures and have received active chemotherapy, and four patients presented with femoral neck fractures as their first presenting symptom of myeloma. For those cases who had been diagnosed with myeloma prior to fracture, the average duration of the disease was 32 months (range: 1–78).

At presentation, all cases had pelvis radiographs and/ or computed tomography (CT) scans, along with long film femoral radiographs for exclusion of acetabular and skip femoral shaft osteolytic lesions. Eight out of the 16 cases had been identified as pathologic fractures secondary to myeloma lesion, in which osteolytic lesions could be recognized in the preoperative radiographs.

**Fig. 1:**
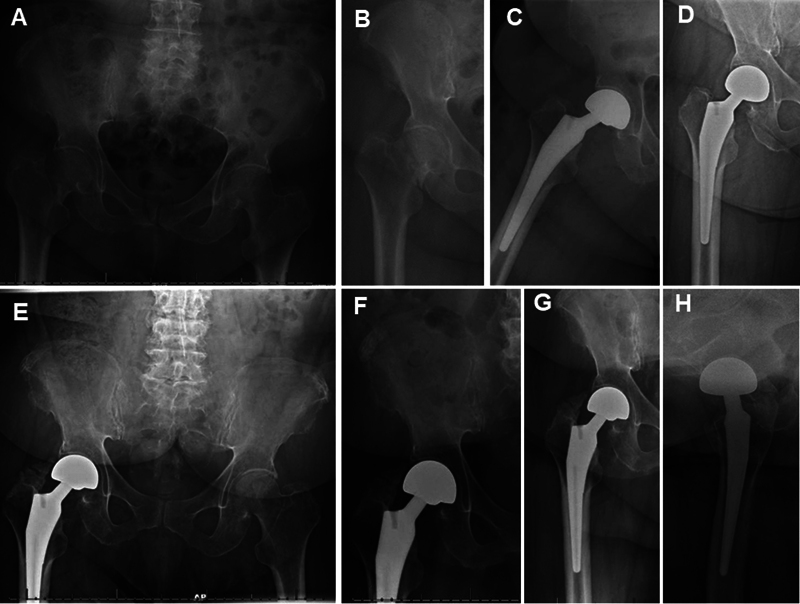
(A, B) Plain pelvic and hip radiographs of a 79.2-year-old female with multiple myeloma showing right sub capital femoral neck fracture. (C) Immediate (same day) postoperative left hip plain X-ray showing cementless bipolar hemiarthroplasty. (D) 6 weeks and (E, F) 56-month, and (G, H) 70-month follow-up plain left hip radiographs showing stable, well-fixed components without evidence of acetabular erosion. Note the presence of grade-III heterotopic ossification.

Fourteen different surgeons performed surgery. Thirteen cases were treated with bipolar HA, and 3 cases were treated with unipolar HA. Surgery was performed through the posterolateral approach in 10 cases while the direct lateral approach was utilized in 6 cases. Fourteen cases received cemented and two received cementless stems. Ten cases received conventional arthroplasty stems, and 6 cases received long stems to bypass skip lesions in the femoral shaft. The average follow-up was 18.2 months (range: 1.3–79.3; Fig. 1 ). None of the cases developed cement implantation syndrome, dislocations, periprosthetic infections, aseptic loosening, or neurovascular injury.

## Complications

### Intraoperative Blood Loss

The average estimated blood loss in all cases was 710 ml (range: 150–2,500 ml). Four cases required intra or postoperative blood transfusion (packed red blood cells/ platelets) without further consequences.

### Periprosthetic Fractures

Three cases (18.75%) were complicated by periprosthetic fractures. Of these three cases, one involved intraoperative fracture of the greater trochanter and was managed with a trochanteric hook plate and cerclage wires. The second case sustained a postoperative greater trochanteric fracture that was evident 3 weeks after surgery and managed nonoperatively. The third case involved a Vancouver type-B distal femur fracture that occurred 3 weeks after surgery due to another low energy fall; this was managed with ORIF with locking plate and cerclage wires. All the fractures occurred in association with cemented stems. To note, the surgical approaches that were utilized during the index HA surgery in the first and third cases was the posterolateral approach, while the second case had the index surgery performed through the direct lateral approach.

### Heterotopic Ossification

Four cases (25%) had grade 2 or 3 heterotopic ossification (HO) involving the acetabulum and/or the trochanteric or subtrochanteric area. All 4 cases were performed through the posterolateral approach. Heterotopic ossification was radiologically evident as early as 6 weeks postoperatively and was managed nonoperatively in both cases.

### Mortality

Thirteen patients (86.6%) died at a mean of 18.5 months (range: 1.3–79.1) after the primary HA surgery. Of them, 3 patients died within the first 3 months of the HA surgery ( Table 2 ). For the patients that passed within 3 months of surgery, the cause of death was acute respiratory failure in 2 patients and septicemia secondary to gastrointestinal infection in one case. The median survival for all cases at the last follow-up or the date of death was 8.4 months.

**Table 2:** Mortality rate in the study group

Mortality at	Number of patients, %
3 months	3; 20%
1 year (+5 patients)	8; 53.3%
5 years (+3 patients)	11; 73.3%

### Acetabular Erosion

**Fig. 2:**
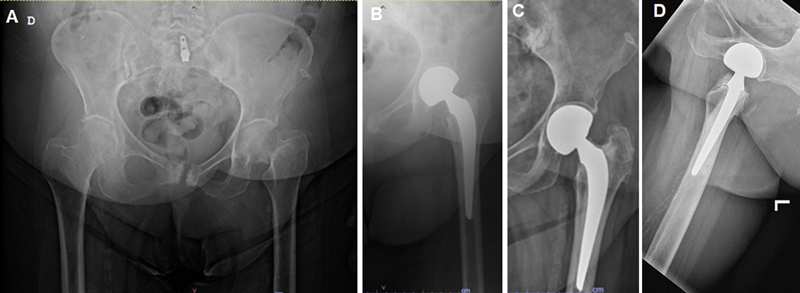
(A) Plain pelvic radiographs of an 82-year-old female showing left displaced femoral neck fracture. (B) Immediate postoperative left hip plain X-ray showing cemented bipolar hemiarthroplasty. (C, D) 16-month postoperative plain left hip radiographs showing acetabular erosion. The patient complained of groin pain with movement and was managed conservatively.

Despite the presence of variable degrees of osteopenia in all of the cases, only 1 case (6.25% of cases), an 82-year-old female ( Fig. 2 ), presented 16 months with mild hip pain with weight bearing after the HA surgery. Plain radiographs suggested mild acetabular erosion. The patient was managed conservatively, and she is alive and under regular follow-up (21 months after the HA surgery).

## Discussion

This is a retrospective series of 16 hip HAs in cases with multiple myeloma. After a mean follow-up of 18 months, 1 case had acetabular wear, 4 cases had radiographically significant heterotopic ossification, and 3 cases had periprosthetic fractures. The 3-month, 1-year, and 5-year mortality rates for this series were 20, 53.3, and 73.3%, respectively.

Patients with pathological fractures secondary to malignant infiltration have a higher risk of perioperative medical complications. 2 13 14 15 When it comes to orthopedic complications, the existing evidence showed conflicting results, since the available literature compared different surgical techniques, for example ORIF, HA, or THA, for different causes of bone infiltration, including primary bone malignancies and metastatic bone infiltration. 4 6 16 17 18 19 20 21 22 23 For example, the life expectancy and the existing comorbidities differ between myeloma and other causes of bony infiltration such as lymphoma, metastatic breast, prostate, or lung cancers. Again, the indications and outcomes of HA differ from the outcomes of ORIF and THA, and the surgical settings are different. 24

Excluding case reports and studies that reported on sporadic cases, the available studies in the literature that reported on hip replacement in myeloma showed different outcomes for different diagnoses and mostly compared several interventions ( Table 3 ). Of them, only two retrospective studies reported solely on a series of myeloma patients. 4 6 Papagelopoulos et al. 4 reported on 53 cases of myeloma who were managed with HA (33 cases) or THA (20 cases). The authors enumerated several complications, including deep infection, dislocation (1.8% each), and a 1-month mortality of 5.6%. The authors did not distinguish whether the complications occurred in HA or THA cases, which are different entities that may be affected by factors such as patients' age, medical comorbidities, activity level, and surgery duration. These factors may be translated to the reported difference in the incidence of periprosthetic infections between HA and THA in the literature, being 1.6 to 10% vs 0.2 to 0.7%, respectively. 25 26 In another study, Park et al. 6 reported on the in-hospital outcomes in 4,011 myeloma cases who received surgery for femoral fractures, extracting the data from a national medical database. In their study, 1,288 patients received HA, 2,555 patients received ORIF, and 168 received THA. When comparing their total outcomes with non-myeloma cases, the authors found that multiple myeloma cases had a higher risk of in-hospital pneumonia, sepsis, surgical site infection, and acute renal failure as well as a lower risk of myocardial infarction. The authors, however, did not distinguish which complications occurred in HA, and which occurred in ORIF and THA.

**Table 3:** Review of the literature regarding the studies that reported about the outcomes of prosthetic replacement in a series of myeloma patients (excluding case reports)

Study/Year	Diagnosis (number of cases)	Intervention (number of cases)	Complications in myeloma cases (number of cases)	Notes
**Papagelopoulos et al. 1997** 4	Multiple myeloma (53)	HA (33) THA (20)	Intraoperative severe blood loss (1) Intraoperative pulmonary distress (1) 1-month mortality (3) Aseptic loosening (1) Dislocation (1) Deep Infection (1) Superficial infection (1) Persistent wound drainage (4)	Two revisions, resection arthroplasty for infection or dislocation (1 each) The authors did not distinguish whether the complications occurred in HA or THA cases.
**Schneiderbauer et al. 2005** 7	Multiple myeloma (31) Other tumors (298)	Hemiarthroplasty	Patients who underwent HA for tumor lesions had a significantly higher incidence of dislocation compared to other indications.	The authors did not mention the number of complications in myeloma cases.
**Parker et al., 2011** 8	Multiple myeloma (9) Other tumors (136)	HA, THA, or ORIF	Patients with Myeloma had the longest mean survival, compared to lymphoma, breast, lung and prostate metastatic lesions.	The authors did not mention the number of complications in myeloma cases, nor the types of interventions.
**Alvi et al., 2013** 9	Multiple myeloma (17) Other tumors (79)	Shoulder Arthroplasty, ORIF, THA and HA (not mentioned)	One case, managed with cemented long stem calcar replacement HA had radiological myeloma disease progression noticed 4 months after the index surgery.	The authors have not mentioned how many of the patients received which implant.
**Park et al., 2016** 6	Multiple myeloma (4,011), comparing in hospital complications with non-myeloma cases.	ORIF (2,555) Hemiarthroplasty (1,288) THA (168)	Patients with multiple myeloma had a higher risk of in-hospital pneumonia (5.4%), sepsis (1.4%), surgical site infection (2.8%), and acute renal failure (9.6%). While there was no difference in DVT, pulmonary embolism, respiratory failure, and in-hospital mortality, multiple myeloma patients had less incidence of myocardial infarction.	The authors did not distinguish which complications occurred in HA and which occurred in ORIF and THA.
**Peterson et al., 2016** 10	Myeloma (5) Other metastatic lesions (17)	Long stem hemiarthroplasty	Sciatic nerve injury (1) Stroke (1) Periprosthetic fracture (1) 1-month mortality (1) 1-year mortality (2)	1 revision for peri prosthetic fracture
**Stevenson et al., 2018** 11	Myeloma (6) Other tumors (94)	Proximal femoral replacement HA (6)	No dislocations, No revisions for pain or acetabular erosion.	The series reported four infections and three revisions; however, the authors did not distinguish whether the complications occurred in myeloma or other cases.
**Seglam et al., 2019** 12	Multiple myeloma (19) Other tumors (114)	Proximal femoral replacement HA (18) Proximal femoral replacement HA (1)	15 patients expired at a mean follow up of 35.11 ± 22.38 months. 12 patients had class 1 or 2 radiological acetabular erosion.	Two revisions, for aseptic loosening and for dislocation (1 each).
**Schwarzkopf et al., 2019** 13	Myeloma (15) Other cases (359 cases)	HA and THA	11/15 myeloma cases had bone cement implantation syndrome, without intraoperative death.	The authors did not distinguish whether complications occurred in HA or THA cases.
**Hayden et al., 2021** 14	Multiple myeloma (25) Other tumors (139)	HA (15) THA (10)	The authors did not find significant differences in the incidence of complications between THA and HA.	The authors did not mention the number of complications in Myeloma cases.

Abbreviations: DVT, deep vein thrombosis; HA, hemiarthroplasty; ORIF, open reduction and internal fixation; THA, total hip arthroplasty.

Other studies included myeloma and non-myeloma cases, reporting on the incidence of complications amongst the total study cohorts ( Table 3 ). To the best of our knowledge, the current study is the first to report solely on the outcomes of hip hemiarthroplasty in patients with myeloma. Despite the small series, the most interesting findings are the high mortality (20% for the 3-month mortality), high incidence of periprosthetic fractures (18.75%), and the relatively low incidence of clinical or radiological acetabular erosion, given the associated osteopenia and osteoporosis that are usually present in myeloma patients. 1 2 3

In patients with multiple myeloma, the reported 1- and 5-year survival rate is about 85 and 58%, respectively. 27 28 When hip fracture occurs, these numbers seem to significantly decline. In our study, only 46.7% of patients survived past 1 year, and 26.7% survived past 5 years from the surgery, while the median survival of all the cases was 8.4 months. Comparatively, Papagelopoulos et al. 4 reported a median survival of 18 months and a 2- and 5-year survival of 43 and 13%. These figures highlight the high mortality of hip fracture in myeloma patients. In the literature, the most common causes of mortality in myeloma patients are adult respiratory failure, septicemia and renal failure. 28 For the 1-month and in-hospital mortality, the incidence in myeloma and non-myeloma patients tends to be similar. 6 In our study, there was no in-hospital mortality, and the first patient died 1.3 months postoperatively, after discharge from the hospital.

Patients with multiple myeloma have weak and osteoporotic bones. 1 2 3 29 30 This may explain the higher incidence of periprosthetic fractures following prosthetic replacement in our study (3 patients; 18.75%). This agrees with the results of Peterson et al., 18 who reported one periprosthetic fracture (PPF) (20%) in 5 HA myeloma cases and required revision. Despite this, Papagelopoulos et al. 4 did not report any PPF in their series of 53 HA and THA myeloma cases. In our study, all the fractures occurred in association with cemented stems. Since the current study has only 2 cases who received uncemented stems, we could not make a recommendation regarding the impact of stem type on the occurrence of periprosthetic fractures in myeloma patients.

Regarding heterotopic ossification (HO), this complication has been rarely reported in patients with multiple myeloma, most likely since it is a less serious complication. Seraj et al. 31 reported it in one patient with multiple myeloma who underwent right hip internal fixation. Due to the small number and the non-comparative nature of the current study, we could not conclude whether the HO cases in our study were related to myeloma or the HA surgery itself. The incidence of radiologically significant HO in our study is 25%, which is less than the reported incidence of HO in HA in general, which reached 27% in one study. 32

Osteoporosis and osteomalacia are known risk factors for acetabular protrusion in native hips. 33 34 When it comes to acetabular wear after hip arthroplasty, the effect of preexisting osteoporosis is controversial. 35 36 37 The incidence of acetabular wear in the current study (6.25%) is comparable to the reported incidence after hip HA in general, which ranges between 0.7 and 17.2%. 38 39 40 Considering these results, HA remains a viable option for management of proximal femoral fractures in myeloma patients. However, patients' expectations regarding the associated morbidities and mortality should be clearly presented as regard to the presented evidence. The lower survival rates in myeloma patients and in this study could potentially have affected the incidence of acetabular erosion in myeloma patients with such osteoporotic bone.

In myeloma patients undergoing hip HA, surgeons should be aware of the incidence of specific complications, especially the potential risk of increased intraoperative blood loss and perioperative PPFs. It is imperative also for the surgeon to properly assess the acetabulum and the entire femur for the presence of osteolytic lesions using CT scans, long film femur radiographs, and even magnetic resonance imaging, 41 to detect the presence of skip myeloma lesions and, hence, allow the surgeon to choose the suitable implant.

The current study has several limitations. This is a small series and is limited by the non-comparative and retrospective study design. A large multicenter study may be required to better evaluate the outcomes of prosthetic replacement in myeloma patients when compared to the general population.

## Conclusion

Hip HA is a reasonable option for the management of femoral neck fractures in patients with multiple myeloma. Provided that the acetabulum is free from tumor invasion, the incidence of acetabular erosion is relatively small. The surgeons should be aware of the associated high morbidity and mortality in such patients.
