# Supplementary material for: Hip Hemiarthroplasty in Patients with Multiple Myeloma: A Retrospective Case Series and Review of the Literature
Source: Rev Bras Ortop (Sao Paulo). 2025 Jan 16;60(1):s00441792099. doi: 10.1055/s-0044-1792099 (PMC11737911; doi:10.1055/s-0044-1792099)
Supplement: Related texts

# Research article: Hemiartroplastia de quadril em pacientes com mieloma múltiplo: Uma série de casos retrospectivos e revisão da literatura

**Keywords:** colo do fêmur, fraturas do quadril, hemiartroplastia, mieloma múltiplo

## Abstract

**Objetivos** As fraturas do colo femoral em pacientes com mieloma múltiplo são geralmente tratadas com hemiartroplastia ou artroplastia total do quadril de acordo com a presença de infiltração acetabular. Devido à escassez de relatos nesta área, o objetivo deste estudo é revisar os desfechos clínicos da hemiartroplastia do quadril em pacientes com mieloma múltiplo e os resultados e a sobrevida neste subconjunto de pacientes.

**Métodos** Um total de 15 pacientes (16 casos), com idade média de 71,7 anos e portadores de mieloma, foram submetidos à hemiartroplastia de quadril para tratamento de fraturas de colo femoral com luxação. Suas radiografias e dados clínicos foram avaliados. Destes casos, 13 passaram pela hemiartroplastia bipolar e três pela unipolar.

**Resultados** Após um acompanhamento médio de 18,2 meses desde o momento da cirurgia, 3 casos sofreram fraturas periprotéticas (18,75%), 4 casos (25%) tiveram ossificação heterotópica e 1 caso (6,25%) apresentou erosão acetabular. A mortalidade dos pacientes em 1 e 5 anos foi de 53,3 e 73,3%, respectivamente.

**Conclusão** A hemiartroplastia de quadril ainda é uma opção terapêutica viável em pacientes com mieloma. No entanto, a morbidade e a mortalidade podem ser altas nesses pacientes e devem ser compreendidas com sensatez antes da cirurgia para atingir bons resultados.

## Introdução

As discrasias de plasmócitos representam uma ampla gama de doenças, incluindo a gamopatia monoclonal de significado indeterminado (GMSI; doença de Kyle), o plasmocitoma ósseo solitário e o mieloma múltiplo. 1 O mieloma múltiplo é a neoplasia óssea primária mais comum, e geralmente causa fadiga, dor musculoesquelética, fraturas patológicas ou infecção recorrente. 1 2 3 A pelve e o fêmur proximal são sítios comuns de infiltrações osteolíticas causadas por lesões de mieloma solitário ou múltiplo. Essas lesões geralmente se apresentam com dor e fraturas iminentes ou patológicas. 4 5 O tratamento cirúrgico das fraturas do colo do fêmur em pacientes com mieloma inclui hemiartroplastia (HA) ou artroplastia total do quadril (ATQ), dependendo principalmente da presença de acometimento acetabular. 3 4 6

Os desfechos da artroplastia na doença óssea metastática foram bem estudados na literatura. Porém, nenhum estudo avaliou especificamente os resultados da HA no tratamento de fraturas em pacientes com mieloma. Os estudos existentes relataram coletivamente os desfechos de duas ou mais opções terapêuticas, como redução aberta e fixação interna (RAFI), HA e ATQ em indivíduos com mieloma 4 6 ou uma coorte que incluía mieloma e outras causas não mielomatosas de infiltração óssea. 7 8 9 10 11 12 13 14 15 O objetivo deste estudo é relatar os desfechos em uma série de casos de pacientes com mieloma submetidos à HA em um centro de trauma de nível 1 em um esforço de contribuir à escassa evidência existente. Nossa principal questão foi se os pacientes com mieloma teriam alta incidência de complicações, em particular fraturas e desgaste acetabular após a HA. Também fizemos uma revisão da literatura com inclusão de todas as séries de casos de HA relatadas em pacientes com mieloma encontradas na literatura.

## Materiais e Métodos

Após a aprovação do Comitê de Ética, um estudo retrospectivo foi realizado para avaliar todos os pacientes submetidos à HA usando os códigos da *Current Procedural Terminology* (CPT - American Medical Association, Chicago, IL, EUA). Um total de 2.488 casos de HA foram identificados entre janeiro de 1988 e junho de 2023. Todos os casos foram revistos em relação aos nossos critérios de inclusão e exclusão para extrair os dados relevantes para este estudo. Os dados coletados incluíram dados demográficos dos pacientes, informações clínicas e avaliações radiográficas, além de informações detalhadas sobre o acompanhamento e a evolução clínica pós-operatória.

## Critérios de Inclusão

1 - Pacientes submetidos à HA para fratura de quadril, principalmente após trauma ou para tratamento de pseudoartrose de fratura do colo do fêmur.
2 - Pacientes com diagnóstico de mieloma múltiplo ou plasmocitoma ósseo.

## Critérios de Exclusão

1 - Pacientes sem notas de acompanhamento clínico após a HA.

## Resultados

Um total de 18 casos (17 pacientes) com discrasias de plasmócitos e submetidos à HA para tratamento de fraturas do colo do fêmur foram recuperados de nosso banco de dados. Dois pacientes foram excluídos por terem GMSI, deixando 16 casos (15 pacientes) inclusos neste estudo.

Destes pacientes, 9 eram mulheres e 6 eram homens, com idade média de 71,7 anos (intervalo: 39,2–89,6) no momento da HA primária. Uma mulher teve fraturas bilaterais do colo do fêmur que ocorreram com 2 meses de intervalo e foi submetida a uma HA unipolar cimentada bilateral. Quatorze pacientes (15 casos) tinham mieloma múltiplo e um apresentava plasmocitoma ósseo solitário no momento da fratura que progrediu para mieloma múltiplo mais tarde. Além das discrasias de plasmócitos, 10 pacientes (11 casos) tinham comorbidades médicas significativas, frequentemente combinadas, como mostra a Tabela 1 . O índice de massa corporal (IMC) médio de 12 pacientes foi de 32,9 (intervalo: 22–52,2), mas não estava disponível nos prontuários de 3 pacientes.

**Tabela 1:** Comorbidades médicas nos pacientes do estudo

Doença renal crônica	4 (1 DRET)
Diabetes mellitus	2
Insuficiência cardíaca	2
Obesidade grave ou mórbida (IMC ≥ 35)	3
Hipotireoidismo	1
Doença de Parkinson	1

Abreviaturas: DRET, doença renal em estágio terminal; IMC, índice de massa corporal.

Todos os casos foram diagnosticados como fraturas do colo do fêmur secundárias a traumas de baixa energia (quedas ao caminhar). Nove pacientes foram diagnosticados com mieloma múltiplo antes da fratura e receberam quimioterapia ativa, enquanto quatro pacientes apresentaram fraturas do colo do fêmur como seu primeiro sintoma de mieloma. Nos casos diagnosticados com mieloma antes da fratura, a duração média da doença foi de 32 meses (intervalo: 1–78).

À apresentação, todos os casos tinham radiografias e/ou tomografias computadorizadas de pelve, além de radiografias femorais de filme longo para exclusão de lesões osteolíticas descontínuas do acetábulo e do eixo femoral. Oito dos 16 casos foram identificados como fraturas patológicas secundárias ao mieloma, e as lesões osteolíticas puderam ser reconhecidas nas radiografias pré-operatórias.

Quatorze cirurgiões diferentes realizaram as cirurgias. Treze casos foram tratados com HA bipolar e três casos com HA unipolar. A cirurgia foi realizada pela abordagem posterolateral em 10 casos, enquanto a abordagem lateral direta foi utilizada em 6 casos. Quatorze casos receberam hastes cimentadas e dois, hastes não cimentadas. Dez casos foram tratados com hastes convencionais de artroplastia e seis com hastes longas para contornar lesões descontínuas na diáfise femoral. O acompanhamento médio foi de 18,2 meses (intervalo: 1,3–79,3; Fig. 1 ). Nenhum dos pacientes desenvolveu síndrome de implantação do cimento, luxações, infecções periprotéticas, afrouxamento asséptico ou lesão neurovascular.

**Fig. 1:** (A, B) Radiografias simples de pelve e quadril de uma mulher de 79,2 anos com mieloma múltiplo mostrando fratura do colo femoral subcapital direito. (C) Radiografia simples de quadril esquerdo no período pós-operatório imediato (mesmo dia) mostrando hemiartroplastia bipolar sem cimento. (D) Radiografias simples de quadril esquerdo em 6 semanas, (E, F) 56 meses e (G, H) 70 meses de acompanhamento mostrando componentes estáveis e bem fixados, sem evidência de erosão acetabular. Observe a presença de ossificação heterotópica de grau III.

## Complicações

## Perda Sanguínea Intraoperatória

A perda média estimada de sangue em todos os casos foi de 710 mL (intervalo: 150–2.500 mL). Quatro casos necessitaram de transfusão de sangue intra ou pós-operatória (concentrado de hemácias/plaquetas) sem consequências posteriores.

## Fraturas Periprotéticas

Três casos (18,75%) apresentaram fraturas periprotéticas como complicação. Destes três, um caso envolveu uma fratura intraoperatória do trocanter maior e foi tratado com uma placa de gancho trocantérica e fios de cerclagem. O segundo caso sofreu uma fratura trocantérica maior pós-operatória que ficou evidente 3 semanas após a cirurgia e foi submetida a tratamento conservador. O terceiro caso foi uma fratura distal do fêmur tipo B de Vancouver, 3 semanas após a cirurgia devido a outra queda de baixa energia, e seu tratamento incluiu RAFI com placa de bloqueio e fios de cerclagem. Todas as fraturas ocorreram em pacientes com hastes cimentadas. É interessante notar que as abordagens cirúrgicas utilizadas na HA índice no primeiro e terceiro casos foram posterolaterais; no segundo caso, a abordagem foi lateral direta.

## Ossificação Heterotópica

Quatro casos (25%) apresentaram ossificação heterotópica (OH) de grau 2 ou 3 com acometimento do acetábulo e/ou da área trocantérica ou subtrocantérica. Todos os quatro casos foram realizados pela abordagem posterolateral. A OH foi radiologicamente evidente já às 6 semanas após a cirurgia e submetida ao tratamento conservador em ambos os casos.

## Mortalidade

Treze pacientes (86,6%) faleceram em uma média de 18,5 meses (intervalo: 1,3–79,1) após a HA primária. Destes, 3 vieram a óbito nos primeiros 3 meses após a HA ( Tabela 2 ). Nestes três indivíduos, a causa da morte foi insuficiência respiratória aguda em dois pacientes e septicemia secundária à infecção do trato gastrintestinal em um caso. A sobrevida mediana de todos os casos no último acompanhamento ou data de óbito foi de 8,4 meses.

**Tabela 2:** Taxa de mortalidade no grupo de estudo

Mortalidade em	Número de pacientes, %
3 meses	3; 20%
1 ano (+5 pacientes)	8; 53,3%
5 anos (+3 pacientes)	11; 73.3%

## Erosão Acetabular

Apesar da presença de graus variáveis de osteopenia em todos os casos, apenas 1 paciente (6,25%), uma mulher de 82 anos ( Fig. 2 ), apresentou dor leve no quadril com sustentação de peso 16 meses após a HA. Radiografias simples sugeriram erosão acetabular leve. A paciente foi submetida ao tratamento conservador e está viva e em acompanhamento regular (21 meses após a HA).

**Fig. 2:** (A) Radiografias pélvicas simples de uma mulher de 82 anos mostrando fratura com luxação do colo do fêmur para a esquerda. (B) Radiografia simples do quadril esquerdo no período pós-operatório imediato mostrando hemiartroplastia bipolar cimentada. (C, D) Radiografias simples do quadril esquerdo 16 meses após a cirurgia mostrando erosão acetabular. A paciente se queixava de dor na virilha com o movimento e foi tratada de forma conservadora.

## Discussão

Esta é uma série retrospectiva de 16 HAs de quadril em pacientes com mieloma múltiplo. Após um acompanhamento médio de 18 meses, 1 caso apresentou desgaste acetabular, 4 pacientes apresentaram OH radiograficamente significativa e 3 casos apresentaram fraturas periprotéticas. As taxas de mortalidade em 3 meses, 1 ano e 5 anos nesta série foram de 20, 53,3 e 73,3%, respectivamente.

Pacientes com fraturas patológicas secundárias à infiltração maligna apresentam maior risco de complicações médicas perioperatórias. 2 13 14 15 Em relação às complicações ortopédicas, as evidências existentes são conflitantes, uma vez que a literatura comparou diferentes técnicas cirúrgicas, como RAFI, HA ou ATQ, em diferentes causas de infiltração óssea, incluindo cânceres ósseos primários e infiltração óssea metastática. 4 6 16 17 18 19 20 21 22 23 A expectativa de vida e as comorbidades existentes, por exemplo, diferem entre o mieloma e outras causas de infiltração óssea, como linfoma ou câncer de mama, próstata ou pulmão metastático. Novamente, as indicações e os desfechos da HA diferem dos resultados da RAFI e da ATQ, assim como os cenários cirúrgicos. 24

Excluindo relatos de casos e estudos de casos esporádicos, a literatura sobre a artroplastia de quadril em pacientes com mieloma mostrou resultados diferentes para diferentes diagnósticos e, em especial, comparou várias intervenções ( Tabela 3 ). Destes, apenas dois estudos retrospectivos relataram uma única série de pacientes com mieloma. 4 6 Papagelopoulos et al. 4 relataram 53 casos de mieloma tratados com HA (33 casos) ou ATQ (20 casos). Os autores enumeraram diversas complicações, incluindo infecção profunda, luxação (1,8% cada) e mortalidade em 1 mês de 5,6%. Os autores não distinguiram se as complicações ocorreram em casos de HA ou ATQ, procedimentos distintos que podem ser afetados por fatores como idade do paciente, comorbidades médicas, nível de atividade e duração da cirurgia. Esses fatores podem ser traduzidos na diferença relatada na incidência de infecções periprotéticas entre HA e ATQ na literatura, de 1,6 a 10% e 0,2 a 0,7%, respectivamente. 25 26 Em outro estudo, Park et al. 6 relataram os desfechos hospitalares de 4.011 casos de mieloma submetidos a cirurgia para tratamento de fraturas femorais, cujos dados foram extraídos de um banco de dados médico nacional. Em seu estudo, 1.288 pacientes foram tratados com HA, 2.555 com RAFI e 168 com ATQ. Ao comparar seus desfechos totais com casos não mielomatosos, os autores descobriram que os pacientes com mieloma múltiplo apresentavam maior risco de pneumonia hospitalar, sepse, infecção do sítio cirúrgico e insuficiência renal aguda e um risco menor de infarto do miocárdio. Os autores, no entanto, não distinguiram quais complicações ocorreram em cada um dos procedimentos.

**Tabela 3:** Revisão da literatura acerca dos estudos que relataram os desfechos da artroplastia em séries de pacientes com mieloma (excluindo relatos de caso)

Estudo/Ano	Diagnóstico (número de casos)	Intervenção (número de casos)	Complicações em pacientes com mieloma (número de casos)	Observações
**Papagelopoulos et al., 1997** 4	Mieloma múltiplo (53)	HA (33) ATQ (20)	Perda sanguínea intraoperatória grave (1) Desconforto pulmonar intraoperatório (1) Mortalidade em 1 mês (3) Afrouxamento asséptico (1) Luxação (1) Infecção profunda (1) Infecção superficial (1) Drenagem persistente da ferida (4)	Duas revisões, artroplastia de ressecção para tratamento de infecção ou luxação (uma cada) Os autores não distinguiram se as complicações ocorreram em casos de HA ou ATQ.
**Schneiderbauer et al., 2005** 7	Mieloma múltiplo (31) Outros tumores (298)	HA	Pacientes submetidos a HA para tratamento de lesões tumorais tiveram uma incidência significativamente maior de luxação em comparação a outras indicações.	Os autores não mencionaram o número de complicações nos casos de mieloma.
**Parker et al., 2011** 8	Mieloma múltiplo (9) Outros tumores (136)	HA, ATQ ou RAFI	Os pacientes com mieloma apresentaram a maior sobrevida média em comparação a linfoma e lesões metastáticas de câncer de mama, pulmão e próstata.	Os autores não mencionaram o número de complicações nos casos de mieloma nem os tipos de intervenções.
**Alvi et al., 2013** 9	Mieloma múltiplo (17) Outros tumores (79)	Artroplastia de ombro, RAFI, ATQ e HA (não mencionado)	Um caso, tratado com HA com haste longa cimentada, apresentou progressão radiológica do mieloma 4 meses após a cirurgia índice	Os autores não mencionaram quantos pacientes receberam qual implante.
**Park et al., 2016** 6	Mieloma múltiplo (4.011); comparação de complicações hospitalares com casos não relacionados a mieloma.	RAFI (2.555) HA (1.288) ATQ (168)	Pacientes com mieloma múltiplo apresentaram maior risco de pneumonia hospitalar (5,4%), sepse (1,4%), infecção do sítio cirúrgico (2,8%) e insuficiência renal aguda (9,6%). Embora não tenha havido diferença em trombose venosa profunda, embolia pulmonar, insuficiência respiratória e mortalidade hospitalar, os pacientes com mieloma múltiplo tiveram menor incidência de infarto do miocárdio.	Os autores não distinguiram quais complicações ocorreram na HA e quais ocorreram na RAFI e na ATQ.
**Peterson et al., 2016** 10	Mieloma (5) Outras lesões metastáticas (17)	HA com haste longa	Lesão do nervo ciático (1) Acidente vascular cerebral (1) Fratura periprotética (1) Mortalidade em 1 mês (1) Mortalidade em 1 ano (2)	Uma revisão para tratamento de fratura periprotética
**Stevenson et al., 2018** 11	Mieloma (6) Outros tumores (94)	HA femoral proximal (6)	Sem luxações, sem revisões por dor ou erosão acetabular.	A série relatou quatro infecções e três revisões; no entanto, os autores não distinguiram se as complicações ocorreram em pacientes com mieloma ou outras doenças
**Seglam et al., 2019** 12	Mieloma múltiplo (19) Outros tumores (114)	HA proximal femoral (18) HA proximal femoral (1)	Quinze pacientes faleceram em um acompanhamento médio de 35,11 ± 22,38 meses. Doze pacientes apresentaram erosão acetabular radiológica de classe 1 ou 2.	Duas revisões por afrouxamento asséptico e luxação (uma cada).
**Schwarzkopf et al., 2019** 13	Mieloma (15) Outros casos (359)	HA e ATQ	11/15 casos de mieloma apresentaram síndrome de implantação de cimento ósseo, sem óbito intraoperatório.	Os autores não distinguiram se as complicações ocorreram nos casos de HA ou ATQ
**Hayden et al., 2021** 14	Mieloma múltiplo (25) Outros tumores (139)	HA (15) ATQ (10)	Os autores não encontraram diferenças significativas na incidência de complicações entre ATQ e HA	Os autores não mencionaram o número de complicações nos casos de mieloma

Abreviaturas: ATQ, artroplastia total de quadril; HA, hemiartroplastia; RAFI, redução aberta e fixação interna.

Outros estudos incluíram casos de mieloma e não mieloma, relatando a incidência de complicações entre as coortes totais ( Tabela 3 ). Até onde temos conhecimento, este estudo é o primeiro a relatar somente os resultados de HA do quadril em pacientes com mieloma. Apesar da pequena série, os achados mais interessantes são a alta mortalidade (20% em 3 meses), a alta incidência de fraturas periprotéticas (18,75%) e a incidência relativamente baixa de erosão acetabular clínica ou radiológica dada a osteopenia e osteoporose geralmente presentes em pacientes com mieloma. 1 2 3

Em pacientes com mieloma múltiplo, a taxa de sobrevida relatada em 1 e 5 anos é de cerca de 85 e 58%, respectivamente. 27 28 A fratura de quadril parece diminuir esses números de maneira significativa. Em nosso estudo, apenas 46,7% dos pacientes sobreviveram mais de 1 ano e 26,7% sobreviveram mais de 5 anos após a cirurgia, enquanto a sobrevida mediana de todos os casos foi de 8,4 meses. Comparativamente, Papagelopoulos et al. 4 relataram uma sobrevida mediana de 18 meses e uma sobrevida em 2 e 5 anos de 43 e 13%. Esses números destacam a alta mortalidade da fratura de quadril em pacientes com mieloma. Na literatura, as causas mais comuns de mortalidade em pacientes com mieloma são insuficiência respiratória adulta, septicemia e insuficiência renal. 28 Em relação à mortalidade em 1 mês e hospitalar, a incidência em pacientes com mieloma e sem mieloma tende a ser semelhante. 6 Em nosso estudo, não houve mortalidade hospitalar, e o primeiro paciente veio a óbito 1,3 meses após a cirurgia, depois da alta hospitalar.

Pacientes com mieloma múltiplo apresentam ossos fracos e osteoporóticos. 1 2 3 29 30 Isso pode explicar a maior incidência de fraturas periprotéticas após a artroplastia em nosso estudo (3 pacientes; 18,75%). Isso condiz com os resultados de Peterson et al., 18 que relataram 1 fratura periprotética (20%) em 5 casos de mieloma submetidos à HA e que necessitaram de revisão. Apesar disso, Papagelopoulos et al. 4 não relataram nenhuma fratura periprotética em sua série de 53 casos de pacientes com mieloma submetidos à HA e ATQ. Em nosso estudo, todas as fraturas ocorreram em indivíduos com hastes cimentadas. Como temos apenas dois casos que receberam hastes não cimentadas, não podemos fazer recomendações sobre o impacto do tipo de haste na ocorrência de fraturas periprotéticas em pacientes com mieloma.

Em relação à OH, esta complicação é raramente relatada em pacientes com mieloma múltiplo, talvez por ser menos grave. Seraj et al. 31 relataram um paciente com mieloma múltiplo que foi submetido à fixação interna do quadril direito e desenvolveu OH. Devido ao pequeno número e à natureza não comparativa do estudo atual, não pudemos concluir se os casos de OH que observamos estavam relacionados ao mieloma ou à HA em si. A incidência de OH radiologicamente significativa em nosso estudo foi de 25%, menor do que a incidência relatada de OH na HA em geral, que chegou a 27% em um estudo. 32

A osteoporose e a osteomalácia são fatores de risco conhecidos para protrusão acetabular em quadris nativos. 33 34 Quanto ao desgaste acetabular após a ATQ, o efeito da osteoporose pré-existente é controverso. 35 36 37 A incidência de desgaste acetabular no estudo atual (6,25%) é comparável à incidência relatada após a HA de quadril em geral, que varia entre 0,7% e 17,2%. 38 39 40 Considerando esses resultados, a HA continua uma opção viável para o tratamento de fraturas femorais proximais em pacientes com mieloma. No entanto, as expectativas dos pacientes em relação às morbidades e mortalidade associadas devem ser claramente apresentadas e baseadas em evidências. As menores taxas de sobrevida em pacientes com mieloma e neste estudo podem ter afetado a incidência de erosão acetabular em indivíduos com ossos osteoporóticos.

Em pacientes com mieloma submetidos à HA de quadril, os cirurgiões devem estar cientes da incidência de complicações específicas, especialmente do risco de aumento da perda sanguínea intraoperatória e fraturas periprotéticas perioperatórias. É também imperativo que o cirurgião avalie bem o acetábulo e todo o fêmur quanto à presença de lesões osteolíticas usando tomografias computadorizadas, radiografias de fêmur de filme longo e até mesmo ressonância magnética 41 para detectar a presença de lesões descontínuas de mieloma e, assim, escolher o implante adequado.

Este estudo tem várias limitações. Esta é uma série pequena e limitada pelo delineamento experimental não comparativo e retrospectivo. Um grande estudo multicêntrico pode ser necessário para maior avaliação dos desfechos da artroplastia em pacientes com mieloma em comparação à população geral.

## Conclusão

A HA do quadril é uma opção razoável para o tratamento de fraturas do colo do fêmur em pacientes com mieloma múltiplo. Desde que o acetábulo esteja livre de invasão tumoral, a incidência de erosão acetabular é relativamente pequena. Os cirurgiões devem estar cientes da alta morbidade e mortalidade em tais pacientes.
